# Prevalence of high-risk human papillomavirus genotypes in outpatient Malian women living with HIV: a pilot study

**DOI:** 10.1186/s12879-024-09412-y

**Published:** 2024-05-22

**Authors:** Ban Traore, Yaya Kassogue, Brehima Diakite, Fousseyni Diarra, Kadidiatou Cisse, Oumar Kassogue, Modibo Diarra, Aissata Coulibaly, Bourama Coulibaly, Hama Diallo, Zoumana Diarra, Madani Ly, Aminata Maiga, Sidi Boula Sissoko, Adama Seydou Sissoko, Cheick Bougadari Traore, Bakarou Kamate, Ibrahima Teguete, Sekou Bah, Guimogo Dolo, Demirkan Besim Gursel, Jane Holl, Lifang Hou, Mamoudou Maiga

**Affiliations:** 1 Centre of Research and Training on Molecular Pathologies, University Hospital of Point G, Bamako, Mali; 2 Faculty of Sciences and Techniques, University of Sciences, Techniques and Technologies of Bamako, Bamako, Mali; 3 Faculty of Medicine and Odontostomatology, University of Sciences, Techniques and Technologies of Bamako, Bamako, Mali; 4 Center of Listening, Care, Animation, and Counseling for People Living With HIV, Bamako, Mali; 5 Faculty of Pharmacy, University of Sciences, Techniques and Technologies of Bamako, Bamako, Mali; 6 Department of Pathology, Feinberg School of Medicine, Northwestern University, Chicago, IL USA; 7 Department of Neurology, University of Chicago, Chicago, IL 60637 USA; 8 Institute for Global Health, Northwestern University, Chicago, IL 60611 USA; 9 Preventive Medicine Department, Northwestern University, Chicago, IL 60611 USA

**Keywords:** Self-sampling, WLWHIV, Hr-HPV, Genotypes, Mali

## Abstract

**Introduction:**

Long-term exposure to high-risk human papillomavirus (Hr-HPV) is a well-known necessary condition for development of cervical cancer. The aim of this study is to screen for Hr-HPV using vaginal self-sampling, which is a more effective approach to improve women’s adherence and increase screening rates.

**Methods:**

This pilot study included a total of 100 Women living with HIV (WLWHIV), recruited from the Center for Listening, Care, Animation, and Counseling of People Living with HIV in Bamako. Hr-HPV genotyping was performed on Self-collected samples using the Cepheid GeneXpert instrument.

**Results:**

The median age of WLWHIV was 44 (interquartile range [IQR], 37–50) years. Approximately 92% of the study participants preferred self-sampling at the clinic, and 90% opted to receive result notifications via mobile phone contact. The overall prevalence of Hr-HPV among study participants was 42.6%, and the most frequent Hr-HPV sub-types observed were HPV18/45 (19.1%), HPV31/35/33/52/58 (13.8%), and HPV39/68/56/66 (12.8%), followed by HPV16 (5.3%), and HPV51/59 (5.3%). WLWHIV under 35 years of age had a higher frequency of Hr-HPV compared to their older counterparts, with rates of 30% versus 11.1% (*p* = 0.03). The duration of antiretroviral treatment showed an inverse association with Hr-HPV negativity, with patients on treatment for 15 (IQR, 10–18) years versus 12 (IQR = 7–14) years for Hr-HPV positive patients (95% CI [1.2–5.8], t = 3.04, *p* = 0.003). WLWHIV with baseline CD4 T-Cell counts below 200 exhibited a higher frequency of Hr-HPV compared to those with baseline CD4 T-Cell counts above 200 (17.9% versus 1.9%, *p* = 0.009). However, other demographics and clinical factors, such as marital status, age of sexual debut, parity, education, history of abortion, history of preeclampsia, and cesarean delivery, did not influence the distribution of Hr-HPV genotypes.

**Conclusion:**

Our findings indicate that WLWHIV under the age of 35 years old exhibited the highest prevalence of Hr-HPV infection, with HPV18/45 being the most prevalent subtype. Additionally, WLWHIV with baseline CD4 T-Cell counts below 200 showed the highest infection rates.

## Introduction

Human papillomavirus (HPV) is one of the most widespread sexually transmitted viral infections, affecting individuals in both industrialized and developing countries. It is known to be associated with the development of several types of cancers [1, 2]. Individuals who have multiple sexual partners or become sexually active at a young age, as well as people with compromised immune systems, are at a higher risk of HPV infection [3]. Fortunately, not all infected individuals will display clinical symptoms due to their strong immune response. However, some may experience genital warts and various types of cancers, including cervical, anal, penile, and throat cancers [4–7]. In addition, pregnant women bearing HPV may undergo or experience preterm labor and give birth to neonates with low birth weight [8]. More than 200 strains of HPV have been identified worldwide; however, 40 genotypes are involved in genital infections [9, 10]. These genitally-tropic HPVs are classified into two main groups. The first group includes high-risk types (16, 18, 31, 33, 34, 35, 39, 45, 51, 52, 56, 58, 59, 66, 68, and 70) that are associated with a higher oncogenic potential [11, 12]. The second group comprises low-risk HPVs (6, 11, 42, 43, and 44) that have a lower likelihood of causing cancer [11, 12]. Numerous studies have demonstrated a higher prevalence of HPV in women with HIV, attributed to the compromised immune response in HIV-infected individuals [13, 14]. Weaker host immunity against HPV in the context of HIV contributes to the development of precancerous lesions and later invasive cancers, in contrast to those with robust immunity [15]. As a result, HIV infection is linked to persistent HPV infection, elevating the risk of specific cancers. Notably, the presence of multiple high-risk HPV types can further increase the risk, especially in women [16, 17]. This relationship between HIV and HPV infections is like a snake biting its own tail. The prevalence of HIV in Mali is around 1.1%, with a total of 110 000 people living with AIDS [18]. Cervical cancer (CC) has been linked to a twofold increase in mortality in WLWHIV [19, 20]. Therefore, identifying of oncogenic HPVs, which are associated with 90% of CC [21] among WLWHIV is a crucial step in the prevention of CC in a resource-limited country like Mali, where HPV vaccination is not yet publicly available. In this study, we proposed a self-sampling method for screening high-risk HPV in women living with HIV to assess both the adherence to the procedure and the prevalence of high-risk HPV in this population. Since 2013, the World Health Organization (WHO) has recommended self-sampling as the preferred technique, which is widely accepted compared to traditional gynecological consultations. Self-sampling overcomes several barriers, such as socio-cultural and religious restrictions for women to be examined by men, embarrassment, privacy concerns, the need for spousal consent, limited understanding of early detection benefits, restricted access to healthcare facilities, lack of screening equipment, and insufficient qualified personal [22, 23]. It allows for the identification of high-risk HPV carriers who can then undergo further assessment and treatment. Consequently, self-sampling is poised to significantly increase CC screening rates and reduce CC-related morbidity and mortality among WLWHIV. To the best of our knowledge, this pilot study represents the first instance of utilizing self-sampling for high-risk HPV prevalence assessment in a Malian cohort of WLWHIV.

## Methods

### Ethical approval and consent to participate

This study received approval from the Ethics Committee of the Faculty of Medicine and Odontostomatology/Faculty of Pharmacy at the University of Sciences, Techniques, and Technologies of Bamako (USTTB) with reference number 2021/205/USTTB. Each participant received a comprehensive explanation of the study procedures, and those who agreed to participate provided informed consent by signing the necessary documents.

### Study participants

WLWHIV undergoing outpatient care were recruited at the Center for Listening, Care, Animation, and Counseling of People Living with HIV in Bamako, Mali, during the period from November 2021 to October 2022. Subsequently, each participant was provided with two self-sampling kits allowing them the choice of conducting vaginal self-sampling either at the clinic in a designated isolation room or in the comfort of their own home. The collected samples were then stored at -20 °C prior to undergoing HPV genotyping. To gather essential information, we designed a data collection form to record demographic, clinical, and biological characteristics of each participant.

### High-risk HPV genotyping

The collected samples were processed within 24 h using Cepheid Reagent Xpert® Xpress MVP Real-Time PCR Molecular Vaginal Panel on Cepheid GeneXpert instrument equipped with four cartridges which enabled the establishment of genotypic profiles of 14 different HPV subtypes. The GeneXpert machine offers six distinct channels for analysis, including P1 for HPV16; P2 for HPV18/45; P3 for HPV 31/35/33/52/58; P4 for HPV 51/59; P5 for HPV 39/68/56/66; and sample adequacy control (SAC). It is important to note that invalid results may occur if a sample does not pass the SAC test, rendering it impossible to determine the presence or absence of HPV target DNA. The positivity cut-off values are set at cycle threshold (Ct) 40 for P1 and P2, while for P3, P4, and P5, the cut-off is Ct 38.

### Management of high-risk HPV positive cases

Within 24 h, study staff called participants back with their HPV test results. Those who tested negative for HPV were invited to resume routine screening according to national guidelines. However, those who were Hr-HPV positive were subjected to perform colposcopy-guided visual inspection of the cervix after application of acetic acid and Lugol’s solution. Furthermore, biopsies were performed by gynecologist on women with suspicious cervixes on visual inspection for histological examinations. Consequently, participants with positive Hr-HPV tests and cervix lesions, indicative of precancerous and cancerous lesion conditions as determined by the pathology department at the University Hospital of Point G, were promptly provided with appropriate treatment and management.

### Statistical analysis

We used SPSS version 16.0, a statistical software by SPSS Inc., Chicago, IL, USA, for data analysis. Descriptive statistics were utilized to determine the frequencies of high-risk HPV (Hr-HPV) among participants. The chi-square (X^2^) test or Fisher’s exact test was used to assess the relationship between participants’ sociodemographic and clinical variables and Hr-HPV genotypes. In the case of continuous variables, the independent sample t-test was applied. A significance level of *p* < 0.05 was considered significant.

## Results

**Fig. 1:**
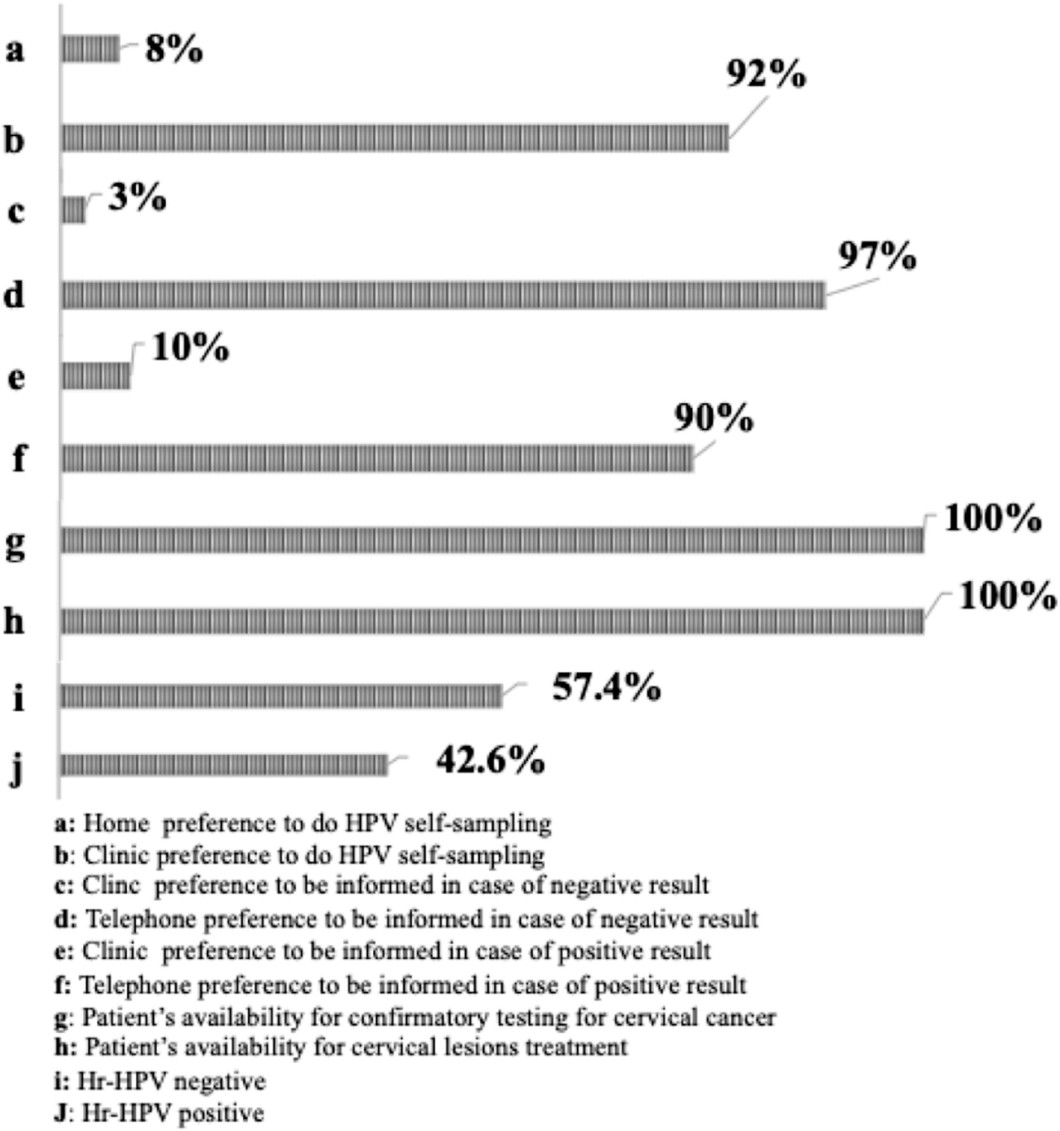
Preferences of women living with HIV regarding the self-sampling procedure, announcement of results, availability for additional testing, and overall distribution of Hr-HPV

**Fig. 2:**
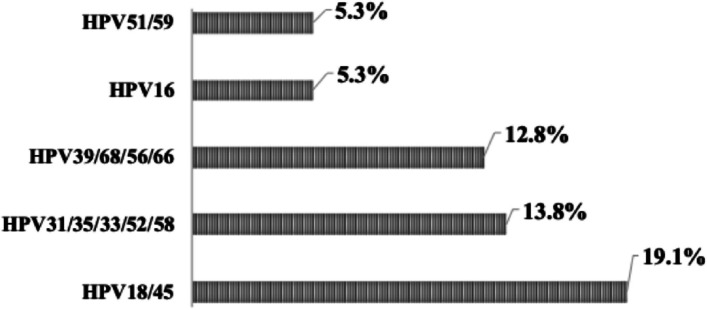
Distribution of Hr-HPV genotypes in women living with HIV

**Fig. 3:**
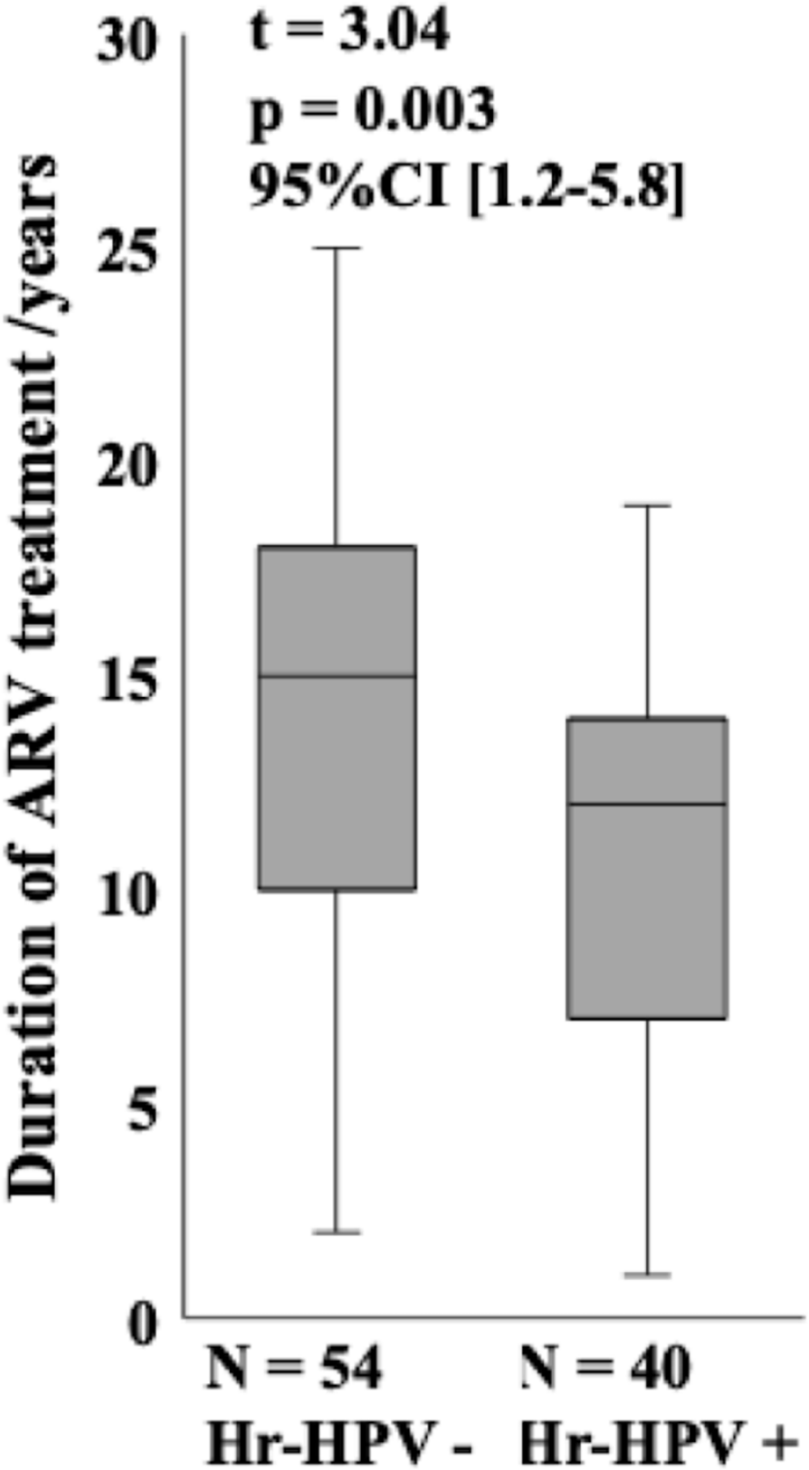
Hr-HPV presence based on the median number of years of antiretroviral therapy

The present study included 100 WLWHIV with a median age of 44 (IQR, 37–50) years. Out of the 100 WLWHIV enrolled, four did not provide samples, thus, Hr-HPV genotyping assay was conducted on 96 self-collected samples, but two samples failed. In total, 94 samples were successfully genotyped and included in the final data analysis. Figure 1 illustrates the participants’ preferences for self-sampling and their willingness to undergo confirmatory tests for cervical lesions and treatment. Notably, 92% of our participants preferred self-sampling at the clinic, while 8% chose to perform it at home. Regarding the notification of negative results, 97% of participants preferred to be contacted via mobile phone, while 3% opted for in-person notification at the clinic. For positive results, 90% chose mobile phone contact, while 10% preferred in-person notification at the clinic. Interestingly, all participants expressed readiness for confirmation and treatment of cervical lesions. Overall, out of the 94 WLWHIV, 40 (42.6%) were positive for Hr-HPV, whereas 54 participants (57.4%) were identified as Hr-HPV negative. Figure 2 displays the distribution of the different Hr-HPV genotypes. The most frequent sub-types were HPV18/45 (19.1%, *n* = 18), HPV31/35/33/52/58 (13.8%, *n* = 13), and HPV39/68/56/66 (12.8%, *n* = 12). HPV16 and HPV51/59 exhibited similar frequencies at 5.3% each (*n* = 5). Table 1 provides a summary of the patients’ sociodemographic and clinical characteristics. Notably, WLWHIV under the age of 35 had a higher prevalence of Hr-HPV carriage compared to those aged 35 years and above, with rates of 66.7% vs. 33.3%, *p* = 0.03. No significant difference was observed in the distribution of Hr-HPV based on marital status, *p* = 0.9. Among sexually active WLWHIV, 50% of those who initiated sexual activity at or before the age of 18 were Hr-HPV-positive, compared to 35.9% for those who began sexual activity after the age of 18 (*p* = 0.3). The frequency of Hr-HPV positivity was 53.7% in monogamous patients versus 34% in polygamous patients, with a *p*-value of 0.06. Concerning parity, 60% of patients with 7–9 children tested positive for Hr-HPV, compared to 38.6% for patients with 4–6 children and 40% for patients with 0–3 children (*p* = 0.3). There was no discernible relationship between education level and Hr-HPV status. Overall, Hr-HPV infection rates were lower among WLWHIV with higher education level (14.8%) compared to those with no education, primary, and secondary levels (43.6%, 44.1%, and 50%, respectively). No significant difference was observed in the distribution of Hr-HPV between patients with a history of abortion and those without (48.4% versus 39.7, *p* = 0.5). The presence of a history of preeclampsia appeared to be associated with an increase likelihood of Hr-HPV carriage (*p* = 0.07). The frequency of Hr-HPV in study participants who had undergone caesarean section was 16.7% versus 46.3% in those with no history of caesarean section, *p* = 0.06. Interestingly, the median time of antiretroviral duration for Hr-HPV-negative patients 15 (IQR, 10–18) years was statistically higher than for Hr-HPV positive patients 12 (IQR = 7–14) years; 95% CI [1.2 − 5.8], t = 3.04, *p* = 0.003 (Fig. 3). In addition, WLWHIV with baseline CD4 T-Cell counts below 200 were more likely to carry Hr-HPV than those with CD4 T-Cell counts above 200 (87.5% versus 36.4%, *p* = 0.008).

**Table 1:** Sociodemographic and clinical features of women living with HIV

Parameters	Hr-HPV+ *N* (%)	Hr-HPV- *N* (%)	X^2^	*p* value
**Age group (years)**				
≤ 35	12 (66.7)	6 (33.3)	5.3	0.03^**^
> 35	28 (36.8)	48 (63.2)		
**Marital Status**				
Married	22 (41.5)	31 (58.5)	0.07	0.9^*^
Divorced	5 (45.5)	6 (54.5)		
Widowed	13 (43.3)	17 (56.7)		
**Sexual debut (years)**				
≤ 18	24 (50)	24 (50)	2.35	0.3*
> 18	14 (35.9)	25 (64.1)		
Missing	2 (28.6)	5 (71.4)		
**Smoking**				
Yes	1 (100)	0 (0)	1.36	0.4^**^
No	39 (41.9)	54 (51.1)		
**Parity**				
0–3	14 (40)	21 (60)	2.2	0.3^*^
4–6	17 (38.6)	27 (61.4)		
7–9	9 (60)	6 (40)		
**Matrimonial regime**				
Monogamy	22 (53.7)	19 (46.3)	3.6	0.06^*^
Polygamy	18 (34)	35 (66)		
**Educational level**				
Unschooled	17 (43.6)	22 (56.4)	2.6	0.4^*^
Primary	15 (44.1)	19 (55.9)		
Secondary	7 (50)	7 (50)		
Higher	1 (14.3)	6 (85.7)		
**Profession**				
Housewife	21 (47.7)	23 (52.3)	2.10	0.3^*^
Seller	14 (43.8)	18 (56.3)		
Other	5 (27.8)	13 (72.2)		
Other STD				
Yes	31 (43.1)	41 (56.9)	0.03	1^**^
No	9 (40.9)	13 (59.1)		
Alcohol consumption				
Yes	2 (66.7)	1 (33.3)	0.7	0.5^**^
No	38 (41.8)	53 (58.2)		
Number of abortion				
0	25 (39.7)	38 (60.3)	0.6	0.5^**^
≥ 1	15 (48.4)	16 (51.6)		
Preeclampsia				
Yes	3 (100)	0 (0)	4.2	0.07^**^
No	37 (40.7)	54 (59.3)		
Cesarean section				
Yes	2 (16.7)	10 (83.3)	3.7	0.06^**^
No	38 (46.3)	44 (53.7)		
CD4 T Cell classes				
≤ 200	7 (87.5)	1 (12.5)	7.4	0.008^**^
> 200	24 (36.4)	42 (63.6)		

*Fisher’s Exact Test

**Pearson Chi-Square

## Discussion

Screening for Hr-HPV, particularly in vulnerable populations like WLWHIV, represents an effective strategy for preventing and managing cervical cancer. In our current pilot study, we used the self-sampling approach, which offers clear advantages in terms of user-friendliness, and the preservation of patient privacy. This method aligns with the goal set by the World Health Organization (WHO), aiming for a 70% HPV screening rate, and this strategy will enable a large number of women to be reached for HPV screening by removing any ambiguity about barriers related to complexity, dignity, religion and culture. Our findings indicated a strong preference among study participants conducting self-sampling at the clinical setting. Similar trends have been observed in previous studies conducted in Botswana and Uganda [24, 25]. The choice of clinic-based self-sampling can be attributed to the WLWHIV desire for confidentiality, the availability of assistance before, during, and after self-sampling, and the convenience of returning self-collected samples. Interestingly, a study conducted in Kenya revealed a different preference, with the majority of WLWHIV opting for self-sampling at home [26]. It is noteworthy that 4 out of the 8 individuals who favored home self-collection returned their samples. This finding has potential implications for future Hr-HPV implementation studies, highlighting the need for proactive measures to address this issue and ensure broad screening coverage. In our study, more than 90% participants chose to receive their Hr-HPV test results via cell phone calls, which proved to be an effective means of encouraging participants to undergo confirmatory testing. This contrasts with the findings of Kohler et al., who reported a lower rate of 47% among WLWHIV in Botswana [24]. Our study revealed that 42.6% (95% CI = 33-52.6%) of WLWHIV were tested positive for Hr-HPV. These findings align with results from several studies conducted across Africa, including Cameroon (46.43%) [27], Botswana (40.4%) [28], South Africa (36.7%) [29], Uganda (45%) [30], Zimbabwe (43%) [31], and Southern Ethiopia (35.2%) [32]. However, a study in Ghana reported a lower frequency of 14.5% [33]. Notably, in Brazil, Rodriguess et al. reported a significantly higher prevalence of Hr-HPV at 77.5% among women living with HIV [34], which contrasts with the prevalence observed in Sub-Saharan Africa. Additionally, in a prior study by Jary et al. conducted in Sikasso (a regional city of Mali), a frequency of 77% for Hr-HPV among WLWHIV tested positive during visual inspection with acid and Lugol’s solution was reported [35]. Our results underscore the presence of Hr-HPV types (35, 56, 59/66) that are not covered by currently available vaccines, even though Mali has not yet implemented a vaccination program. This finding echoes the observations of several other studies reporting a high frequency of non-vaccine HPV types among WLWHIV in Sub-Saharan Africa, described in a systematic review [36]. Thus, our study emphasizes the importance of reinforcing Hr-HPV screening programs as a crucial step in preventing cervical cancer across the Sub-Saharan region, and the prevalence of Hr-HPV in women living with HIV is consistently close to 40%. Numerous global studies have demonstrated that the outcomes of the self-sampling method are comparable to those of health professional-based Hr-HPV screening test [37–40]. Therefore, the self-sampling method holds significant promise for increasing Hr-HPV screening rates in low- and middle-income countries. Our study found that the prevalence of Hr-HPV infection was statistically higher in WLWHIV aged 35 years or younger compared to their older counterparts. This trend mirrors findings in other studies conducted in Kenya, Ivory Coast, and Nigeria [41–43]. Therefore, it is imperative for oncogenic HPV screening programs to place heightened emphasis on this specific age group to detect cervical lesions at an early stage and provide timely and adequate treatment. Regarding other demographic and clinical parameters, including marital status, age of sexual activity initiation, education, parity, abortion history, history of preeclampsia, and cesarean delivery history, no statistically significant differences were observed between Hr-HPV-positive and Hr-HPV negative women living with HIV. This current study results highlighted that a baseline CD4 T-cell count below 200 was significantly associated with a higher frequency of Hr-HPV infection. Similar findings have been documented in studies conducted in the Cameroon and Bahamas populations [27, 44]. Consequently, routine screening for Hr-HPV infection in WLWHIV should be integrated into HIV diagnosis procedures to alleviate the burden of immunosuppression and enable prompt treatment of cervical lesions. Furthermore, our study revealed that a longer duration of antiretroviral treatment was associated with a higher probability of Hr-HPV clearance. This underlines the importance of antiretroviral therapy and patient compliance in boosting the immune system to reduce HPV carriage in this particularly vulnerable population.

## Conclusion

This pioneering pilot study utilizing self-sampling for Hr-HPV screening revealed an overall Hr-HPV distribution among WLWHIV. We noticed that, younger WLWHIV exhibited the highest rate of Hr-HPV infection. Among the Hr-HPV subtypes, HPV18/45 emerged as the most prevalent. Additionally, our findings underscored that the highest infection rates are observed in WLWHIV with low CD4 T-cell counts at baseline. These insights gleaned from our study hold potential relevance for future implementation studies and inform strategies for HPV vaccination programs.

## Data Availability

Data used in this study is not publicly available but can be requested form corresponding author [YK] upon reasonable request.
